# Timber harvesting was the most important factor driving changes in vegetation composition, as compared to climate and fire regime shifts, in the mixedwood temperate forests of Temiscamingue since AD 1830

**DOI:** 10.1007/s10980-025-02043-x

**Published:** 2025-01-20

**Authors:** Daniela Robles, Yan Boulanger, Jesus Pascual, Victor Danneyrolles, Yves Bergeron, Igor Drobyshev

**Affiliations:** 1 Institut de recherche sur les forêts, Université du Québec en Abitibi-Témiscamingue (UQAT), 445 boul. de l’Universite, Rouyn-Noranda, QC J9X 5E4 Canada; 2 Herbario Nacional del Ecuador (QCNE), Instituto Nacional de Biodiversidad, Avenida Río Coca E6-115 e Isla Fernandina, Quito, Ecuador; 3 Centre de Foresterie Des Laurentides, Canadian Forest Service, Natural Resources Canada, 1055 rue du Peps, Quebec City, QC G1V 4C7 Canada; 4 Université du Québec à Chicoutimi, 555 Bd de l’Université, Chicoutimi, QC G7H 2B1 Canada; 5 Centre d’étude de la forêt, Université du Québec à Montréal, Succ. Centre-ville, C.P. 8888, Montreal, QC H3C 3P8 Canada; 6 Southern Swedish Forest Research Centre, Swedish University of Agricultural Sciences, Box 190, 234 22 Lomma, Sweden

**Keywords:** LANDIS-II, Retrospective modeling, Forest disturbances, Vegetation composition shifts, Logging, Fire regime shifts, Climate change, Northeastern North America, Mixedwood temperate forests

## Abstract

**Context:**

The vegetation composition of northeastern North American forests has significantly changed since pre-settlement times, with a marked reduction in conifer-dominated stands, taxonomic and functional diversity. These changes have been attributed to fire regime shifts, logging, and climate change.

**Methods:**

In this study, we disentangled the individual effects of these drivers on the forest composition in southwestern Quebec from 1830 to 2000 by conducting retrospective modelling using the LANDIS-II forest landscape model. The model was run based on pre-settlement forest composition and fire history reconstructions, historical timber harvest records, and climate reanalysis data. We compared counterfactual scenarios excluding individual factors to a baseline historical scenario.

**Results and Conclusions:**

Our results indicated that timber harvesting had the greatest impact on forest dynamics over the past centuries. In the absence of timber harvesting, pre-settlement species abundances were largely maintained, preserving key functional traits like fire and shade tolerance that contribute to ecosystem resilience. Increased fire activity during the settlement period contributed to the increase of early-successional aspen (Populus tremuloides), but timber harvesting played the dominant role. Fire exclusion had no influence on vegetation composition, suggesting mesophication unfolds over longer timescales than those captured in this study. Climate change, characterized by modest increases in temperature and precipitation, had a minor effect on vegetation shifts, as increased precipitation might have mitigated the adverse effects of rising temperatures. However, future climate change is projected to become a more significant driver of forest composition. These findings underscore the importance of forest restoration and continued research on past forest dynamics to better understand current and future changes.

**Supplementary Information:**

The online version contains supplementary material available at 10.1007/s10980-025-02043-x.

## Introduction

Forest vegetation composition is shaped by complex interactions among climate, disturbances, and other environmental factors, operating across multiple temporal and spatial scales (Oliver et al. 1996). While long-term vegetation dynamics at subcontinental scales have been driven by climate over millennia (Williams et al. 2004), abrupt climate change events can disrupt these trends (Shuman et al. 2009), causing rapid shifts in vegetation composition (Williams et al. 2002; Shuman et al. 2004; Yu 2007). Though some taxa may respond more slowly, with time lags extending over centuries (Williams et al. 2002). Disturbances such as wildfires, windstorms, and insect outbreaks also shape forest ecosystems. Disturbances alter ecosystem structure and simultaneously release resources (e.g., growing space, light, and nutrients) that promote recovery and ecological succession (Pickett and White 1985; White and Jentsch 2001). Forests often exhibit resilience to natural disturbance regimes (Johnstone et al. 2016) due to adaptive traits shaped by long-term exposure (Sousa 1984; Keeley et al. 2011). However, disturbances that exceed the historical range of variability can severely impact ecosystems, as the prevailing communities may lack the traits needed to recover under novel conditions (Turner and Seidl 2023). Human land use and climate change have drastically altered disturbance regimes and forest composition, with potentially detrimental effects on ecosystem functioning, including carbon uptake (Ojima et al. 1994; Seidl et al. 2017; Thom et al. 2018; Danneyrolles et al. 2019).

Forests in northeastern North America (northeastern United States and southeastern Canada) have undergone major shifts in vegetation composition from pre-settlement times to the present. Forests dominated by white pine (*Pinus strobus* L.) and red pine (*Pinus resinosa* Ait.) were once widespread across the region. However, in some areas of their range, such as the Great Lakes region, as little as 0.6% of the pre-settlement primary red-white pine forests remains (Frelich 1995; Ziegler 2010). This drastic reduction in pine-dominated forests is part of broader regional vegetation changes. Slow-growing conifers such as pines (*Pinus* spp.), spruces (*Picea* spp.), balsam fir (*Abies balsamea* (L.) Mill.), and white cedar (*Thuja occidentalis* L.) have been largely replaced by fast-growing deciduous species, including poplars (*Populus* spp.), paper birch (*Betula papyrifera* Marsh.), and maples (*Acer* spp.) (Jackson et al. 2000a; Friedman and Reich 2005; Schulte et al. 2007; Dupuis et al. 2011a; Danneyrolles et al. 2016a; Terrail et al. 2019). These shifts contributed to the homogenization of taxonomic and functional diversity (Jackson et al. 2000b; Schulte et al. 2007; Pinto et al. 2008; Dupuis et al. 2011b; Hanberry et al. 2012; Danneyrolles et al. 2016b, 2021).

Frequent low- to moderate-severity surface fire with infrequent stand-replacing fires played a critical role in maintaining pine-dominated forests in the region (Drobyshev et al. 2008). Low- to moderate-severity fires created conditions essential for pine regeneration by reducing the soil organic layer (Nyamai et al. 2014; Stambaugh et al. 2018), increasing light penetration to the forest floor by reducing canopy cover (McRae et al. 1994), and eliminating shade-intolerant competitors (Nyamai et al. 2014; Stambaugh et al. 2018). Occasional stand-replacing fires or other large-scale disturbances such as hurricanes, also favored pines’ regeneration, especially that of white pine, by creating large canopy gaps and reducing competition for light and soil nutrients (Abrams 2001).

Changes in land use have influenced fire regime shifts in eastern North America. During the Euro-American settlement, fire activity increased due to land clearing, agricultural fires, and unintentional ignitions, such as those from railway sparks (Weir and Johnson 1998; Stambaugh et al. 2018; Terrail et al. 2020). Subsequently, fire activity declined due to fuel fragmentation caused by forest conversion to croplands and infrastructure development (Guyette et al. 2002; Stambaugh et al. 2018), and active fire suppression measurements introduced in the 1900s that became increasingly effective by the 1970s (Lauzon et al. 2007; Gauthier et al. 2008; Cardil et al. 2019).

Climate change likely directly influenced the vegetation composition of northeastern North America throughout the late Holocene. The Little Ice Age (LIA; ~ 1450–1850 CE) witnessed an abrupt decline in hardwoods (*Fagus, Acer,* and *Betula*) and the mesophytic *Tsuga*, coupled with an increase in boreal taxa (*Picea glauca* (Moench) Voss and *Abies balsamea* (L.) Mill*.*) (Houle et al. 2012; Paquette and Gajewski 2013). Climate change may have indirectly affected forest composition through its effects on fire regimes. Drought conditions during the 1910s and 1920s could have exacerbated the settlement-related fire activity (Girardin et al. 2004). Changes in atmospheric circulation patterns since the end of the LIA have led to lower drought severity, increased cyclonic activity, and the influx of moist air masses (Girardin et al. 2006), contributing to the lengthening of fire cycles in eastern North America (Drobyshev et al. 2017; Chavardès et al. 2022). Vegetation changes observed since the settlement period might, therefore, be a continuation of climate-mediated dynamics driven by large-scale changes in atmospheric circulation regimes (Gajewski et al. 1985; Houle et al. 2012; Paquette and Gajewski 2013).

Forest harvesting has been an important factor affecting vegetation composition in the region. Commercial logging in northeastern North American forests progressed from the extraction of square timber to sawn timber and ultimately pulpwood. Historical logging practices, including diameter-limit cutting and clear-cutting, considerably degraded these forests (Kenefic et al. 2005; Archambault et al. 2009). Selective cutting of large-diameter pines was particularly damaging as it left behind large volumes of surface and ladder fuels that favored the spread of surface fires into crowns, killing canopy pines (Lower 1933; Whitney 1987). Clear-cutting supported the invasion of fast-growing species, such as trembling aspen (*Populus tremuloides* Michx., hereafter aspen) (Graham et al. 2011), and decreased the proportion of coniferous to deciduous species (Archambault et al. 1998).

Despite the recognized impacts of climate change, changing fire regimes, and timber harvesting on the vegetation composition, their specific impacts on forest dynamics over recent centuries remain unclear (Abrams and Nowacki 2015; Nowacki and Abrams 2015; Liang et al. 2018, 2023; Brice et al. 2019, 2020; Danneyrolles et al. 2019). The variation in the spatial and temporal resolution of available historical data makes it challenging to isolate the individual effects of each driver. However, assessing their individual contributions to the dynamics of single species is critical for development of informed conservation and management strategies. To address this knowledge gap, we conducted a retrospective modeling analysis using LANDIS-II, a spatially explicit forest landscape model. This approach enabled hypothesis-testing of paleoenvironmental change (Berland et al. 2011; Klimaszewski-Patterson et al. 2018) and the possibility to distinguish the relative importance of the driving factors in vegetation shifts. We examined the Temiscamingue region of southwestern Quebec as a case study due to its well-documented historical ecology and the availability of historical data. We used (a) pre-settlement vegetation reconstructions to define the initial vegetation composition for the simulations, (b) fire regime reconstructions, (c) historical timber harvest data, and (d) climate reanalysis data from the pre-settlement times to the present. We simulated vegetation dynamics and forest disturbances from 1830 to 2000 (170 years). We tested three hypotheses: (H1) timber harvesting had a greater impact on vegetation composition than fire regime shifts and climate variability. This is because logging represented a more substantial and immediate disturbance compared to fire regime shifts and particularly to the modest climate change observed during the study period; (H2) without timber harvesting, a strong driver, the ecosystem would have maintained its resilience by preserving pre-settlement species composition, and thus a diverse range of key functional traits; and (H3) the decline in fire activity since 1940, due to climate change and fire suppression, had a greater effect upon forest composition than the increased fire activity associated with the settlement period (1890–1940). By elucidating the distinct roles of climate change, fire activity, and forest harvesting on the vegetation dynamics of mixedwood temperate forests, our study provides critical insights to understand past and future dynamics to develop adapted conservation and management strategies.

## Methods

### Study area

**Fig. 1:**
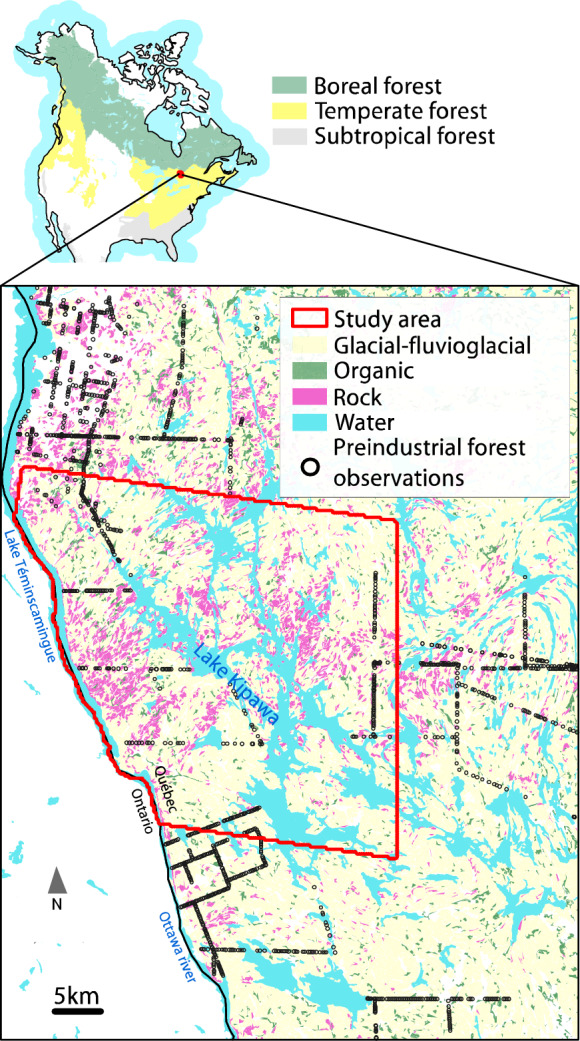
Pre-industrial species lists point observations (black circles and lines, *N* = 1474) located within our study area (perimeter shown as red-outlined polygon) and within a 20 km buffer area surrounding the study area that we used to populate our initial vegetation landscape raster based on the surface deposit class

The study area extended over 1300 km^2^ in the Temiscamingue region in southwestern Quebec (Fig. 1). The area included the Opemican National Park (established in 2018), bordering the Temiskaming Lake on the west and the western parts of the Kipawa Lake on the east. The study area is within the Grenville geological province of the Canadian Shield, and its surficial deposits are primarily clays deposited by the pro-glacial Barlow Lake and rocky glacial till (Vincent and Hardy 1977; Brown 1981). The mean annual temperature in the area is 3.1 °C; with January and July having average temperatures of −15 °C and 18.3 °C, respectively. Average annual precipitation is 836.5 mm, according to the closest weather station in Ville Marie.

The study area at the northern edge of the sugar maple–yellow birch bioclimatic domain of southwestern Quebec (Saucier et al. 1998), which is part of the broader Great Lakes-St. Lawrence forest region of eastern Canada (Rowe 1972). Common tree species found in the study area include sugar maple (*Acer saccharum* Marsh.), yellow birch (*Betula alleghaniensis* Britt.), eastern hemlock (*Tsuga canadensis* (L.) Carr.), white birch (*Betula papyrifera* Marsh.), aspen, large tooth aspen (*Populus grandidentata* Michx.), and fire-associated species such as red pine and white pine occurring in the more xeric sites.

Before the settlers’ arrival, Temiscamingue was inhabited by various nomadic indigenous groups who were primarily part of the Algonquian language family (Riopel 2002). The impact of these Native American groups on the forests of Temiscamingue is not well understood. Nonetheless, research conducted in southern Ontario suggested that Native American use of fire had significant effects on the pre-settlement forests (Clark and Royall 1995; Munoz and Gajewski 2010). However, this perspective has been a subject of debate among other scholars (Campbell and McAndrews 1995; Munoz et al. 2014; Danneyrolles et al. 2016a).

The Euro-American colonization began in 1885, facilitated by the construction of the railway in 1890 (Ville Temiscaming 1996; Riopel 2002). Forestry played a vital role for settlers and progressed through three phases with the focus on square timber, sawn timber, and pulpwood. The emergence of a new timber product did not necessarily replace the previous one but rather coexisted alongside it. Square timber harvesting, which started around 1860 (Riopel 2002) and ended in 1908 (Aird 2016), targeted white and red pine larger than 50 cm in diameter (Lorimer 2008). Sawn timber production began in 1887 (Ville Temiscaming 1996), with companies primarily using white pine, red pine, spruce, and birch (Gourd 1983). The pulpwood industry began in 1918 (Ville Temiscaming 1996). Logging operations gradually expanded northward into Abitibi by the 1920s, and by the mid-twentieth century, nearly half of the harvested wood came from this region (Gourd 1983). Pulpwood primarily used black spruce (*Picea mariana* (Mill.) B.S.P.), balsam fir (*Abies balsamea* (L.) Mill.), hemlock, and aspen (Howe 1923).

### Data

#### Climate data

The climate data we used were spatially downscaled monthly time series from the Twentieth Century Reanalysis version 3 (20CRv3), an ensemble that provides sub-daily global atmospheric conditions from 1836 to 2015 with a spatial resolution of 1° (Slivinski et al. 2019). These reanalyses indicate that since 1836, the mean annual temperature in the study area has increased by 0.47 °C, while precipitation has modestly risen by 15.29 mm (1.66%). To increase the spatial resolution of the 20CRv3 and reflect local variation in climate within the simulations, we used BioSIM, a software that allowed us to interpolate the georeferenced climate data to our landscape grid cells, adjusting for differences in latitude, longitude, and elevation using spatial regressions (Régnière 1996). We used the spatially downscaled average climate data for the period 1981–2010 as a reference and adjusted these data to reflect different average climate conditions for each 30 year period starting from 1836 (the first period 1836–1860 is 24 instead of 30 years). We used 30 year periods as this is the typical duration used in climate studies to provide robust estimates of average climate conditions (WMO 2017). This adjustment involved calculating the difference, or the “delta”, between the average conditions for the reference period (1981–2010) and the average conditions of each 30 year periods: 1836–1860, 1861–1890, 1891–1920, 1921–1950, and 1951–1980. We then adjusted the spatially downscaled average climate data of the reference period (1981–2010) by subtracting the deltas of each period to estimate historical climate conditions for each 30 year period.

#### Historical forest composition data

We obtained the vegetation data from pre-settlement reconstructions of forest composition, developed from land surveys of forest concessions conducted in Temiscamingue starting from the 1850s (Danneyrolles et al. 2016b). The data consisted of species lists derived from surveyors’ logbooks associated with georeferenced points along surveyed transects. We obtained 1474 of these georeferenced pre-settlement vegetation plots located both within our study area and within a 20 km buffer surrounding it (Fig. 1).

#### Fire regime data

We obtained the fire data from fire cycle reconstructions developed for the area from dendrochronological techniques and archival data from provincial and national government sources (Grenier et al. 2005). Our study period encompassed three distinct fire regimes: pre-settlement (1830–1890), settlement (1890–1940), and modern (1940–2000). The reconstructions indicate that the average fire cycle for the pre-settlement period was approximately 262 years, while for the settlement period it was about 96 years (Grenier et al. 2005). In contrast, the modern fire cycle is longer than 3000 years (Boulanger et al. 2014).

#### Timber harvest data

We obtained the harvest data from the annual reports of the commissioners of the Crown lands for Quebec, which later became the Ministry of Lands and Forests of Quebec, retrieved from the Library of the National Assembly of Quebec (https://www.bibliotheque.assnat.qc.ca//fr/). We used data for the Upper Ottawa Valley region because Temiscamingue is located within the broader Upper Ottawa region, and data specifically for Temiscamingue were unavailable in these reports. We assumed that the extracted volume per surface area unit for each species in the Upper Ottawa valley was uniform across the region. Despite some inconsistencies and necessary assumptions, these reports present the most comprehensive and official source of information available for historical logging.

**Table 1:** Timber extracted in tons over the whole landscape

Period	Logging type	Sampling rule	Species	Min. age	Extracted mass (tons)
1830–1859					
1860–1889	Selective		Pinus strobus	200	96,354
1860–1889	Selective		Pinus resinosa	150	14,021
1890–1929	Diameter-limit		Pinus strobus	200	27,324
1890–1929	Diameter-limit		Pinus resinosa	150	97
1890–1929	Diameter-limit		Pinus strobus	70	127,889
1890–1929	Diameter-limit		Pinus resinosa	130	177,188
1890–1929	Diameter-limit	presence of > 3 mature species	Picea glauca	50	131,384
			Abies balsamea	50	
			Tsuga canadensis	60	
			Betula alleghaniensis	50	
			Populus tremuloides	80	
1930–1989	Clear-cut		all species	60	
1990–2000	Partial-cut		all species	60	

The values for the square timber and sawn timber were obtained from the annual reports of the commissioners of the crown lands for Quebec. These reports documented the volumes extracted for species for different types of timber for the whole upper Saint Laurence region. We assumed that our landscape was within this region and the extraction occurred uniformly across the region. We converted volumes to mass in tons using the green wood density of each species

**Table 2:** Summary statistics of the classification of the landscape’s raster cells based on three surface deposit classes: fluvioglacial or glacial, organic, and rock types

Group	Number of cells	Surface deposit	Altitude (in meters)			Slope (in degrees)		
			Range	Mean	SD	Range	Mean	SD
1	11,257	Fluvioglacial or glacial	153–373	295.45	27.21	0–26.28	2.35	2.38
2	769	Organic	165–364	283.11	30.18	0–11.11	1.86	1.80
3	2961	Rock	147–389	288.10	32.40	0–24.86	2.66	2.74
0	5723	Water						

We distinguished four distinct timber harvesting phases: (a) selective cutting of large white and red pines for square timber during 1860–1910; (b) diameter-limit cutting of white and red pines, and cuttings of at least three species among white spruce, balsam fir, eastern hemlock, yellow birch, and aspen for sawn timber during 1890–1930; (c) clear-cutting of all species during 1930–1990 and (d) partial-cutting of all species during 1990–2000. Species-specific extracted volumes and minimum age information for each phase are summarized in Table 1. Detailed descriptions of timber harvest data processing, including diameter limits, volume conversions, and assumptions made are provided in Supplementary Information (SI) 1.

### Modelling approach

Since we worked with a historical landscape, our retrospective simulations relied heavily on proxies we developed given that historical datasets are not as detailed as the modern ones. This was particularly the case for the initial vegetation conditions and harvesting data. For our simulations, we used LANDIS-II, a spatially explicit raster-based forest model that simulates stand- and landscape-level processes. At the stand-scale, LANDIS-II simulated establishment, competition, growth, and mortality based on species-specific life history traits. At the landscape-scale, LANDIS-II simulated seed dispersal and disturbances (Scheller et al. 2007). In the model, the landscape was represented as a grid of interacting cells each containing species-age cohort information. Cells were aggregated into spatial units, termed *landtypes*, with homogenous climatic and edaphic conditions.

LANDIS-II has been used extensively to study the effect of disturbances and their interactions on the vegetation composition under climate change (e.g., Lucash et al. 2018; Molina et al. 2021, 2022). However, only a few studies have used it for retrospective analyses (Berland et al. 2011; Klimaszewski-Patterson et al. 2018; Wu et al. 2022). Building upon their methodologies, we utilized landscape modeling to create scenarios to investigate past landscape dynamics, enabling hypothesis testing on the drivers of past forest composition change.

### Model parameterization

Our study area was represented as a grid of 20,800 cells, each with an area of 6.25 ha (250 × 250 m^2^). To classify each cell in our landscape to a landtype, we used the national soil property maps for Canada (Mansuy et al. 2014). We classified our study area into three landtypes (Table 2): fluvioglacial and glacial (11,257 cells), organic (769), and rock (2961). The remaining 5723 cells were water. We ran the simulations in 10 year time steps.

#### Initial vegetation conditions

Since it was impossible to retrieve an exact map of the pre-settlement forest composition, we employed a random assignment approach to generate the initial vegetation map representing the pre-settlement vegetation cover. This approach relied on randomly assigning a georeferenced pre-settlement vegetation plot (*N* = 1474) (Fig. 1, Historical forest composition data section above) to each cell in our landscape raster based on the matching characteristics of their surface deposit classes. In cases where the species lists provided only the genus-level identification (*Picea*, *Pinus*, *Acer*, *Populus*, and *Fraxinus*), we randomly assigned one or the other or both (33.33–33.33–33.33% probability) of the two most common species within the taxonomic group that are found in our study area. If the reconstructed vegetation plot indicated the presence of *Picea* spp., we assigned to the plot either *Picea glauca*, *P. mariana*, or both. For *Pinus* spp., we assigned *Pinus resinosa*, *Pinus strobus*, or both. For *Acer* spp., we assigned *Acer rubrum*, *Acer saccharum*, or both. For *Populus* spp. and *Fraxinus* spp., we kept them aggregated as the individual species of these genera do not vary considerably in their life-trait properties in this area.

To assign an age structure to the landscape and establish age cohorts for all forested cells, we conducted spin-off simulations for 1000 years using the Base Fire extension of LANDIS-II (He and Mladenoff 1999). The Base Fire extension can simulate stochastic fire events based on a few parameters such as fire size, fire spread and ignition. Simulations were conducted using variations around the pre-settlement fire cycle by varying the *k* parameter, which determines the return interval based on the rate of accumulation of combustible materials, and the probability of ignition. In particular, we tested various reconstructed pre-settlement fire cycles within the estimated range of 141–519 years (95% confidence interval) for this region (Grenier et al. 2005). Fire size distribution was based on the one described under the current climate by Boulanger et al. (2014). We selected the combination of fire cycle and fire parameters that (a) preserved the presence of all initial species without causing extinctions, and (b) maintained the vegetation proportions consistent with the simulation’s initial state. Based on these criteria, the 300 year fire cycle was determined to be the most suitable. For the climate, we used the spatially downscaled average climate conditions for the period of 1836–1860 for our study area (see Climate data section above) and kept these conditions constant for the whole duration of the spin-off simulation.

#### Climate change impacts on stand-level forest dynamics

To account for climate change impacts on stand-level forest dynamics from the pre-settlement to the present time, we used the Biomass Succession extension for LANDIS-II (Scheller and Mladenoff 2004). This extension models changes in cohort aboveground biomass (AGB) over time by accounting for tree species’ cohort age, life-history traits, and species-specific responses to different landtypes. We gathered life-history trait data for species from various sources, including numerous past LANDIS-II studies on North American forest landscapes. We parameterized and calibrated three sets of dynamic inputs that respond to soil and climate conditions: (i) species establishment probabilities (SEP), (ii) maximum potential aboveground net primary productivity (maxANPP), and (iii) maximum aboveground biomass (maxAGB). This parameterization was performed using the individual tree-based forest patch model PICUS version 1.5 (Lexer and Hönninger 2001; Taylor et al. 2017). PICUS models the dynamics of individual trees within 10 × 10 m patches across forest stands and incorporates spatial interactions among patches using a 3D light module. It also simulates the effects of climate and soil characteristics on tree population dynamics (Lexer and Hönninger 2001). We utilized PICUS simulations with species-specific parameters for different tree species present in the study areas. To determine the three dynamic input parameters for the Biomass Succession extension, we conducted PICUS simulations of mono-specific 1-hectare stands for each of the tree species. A factorial design approach was used, simulating mono-specific stands by species and landtype under varying climate conditions across each landtype every 30 years. The simulations were run over 300 years, beginning from bare-ground, utilizing landtype-specific soil data and climate data corresponding to each period. Values for SEP, maxANPP, and maxAGB were extracted from these simulations following (Boulanger et al. 2017).

#### Fire regime shifts

We simulated changes in fire regime using the Base Fire extension (He and Mladenoff 1999). Based on the historical fire regime parameters, we calibrated the extension by modifying the *k* and then the *p* parameters obtained from the spin-off exercise so that their combination would result in the historical fire cycles considered (see Fire regime data section above) at ± 10%. Fire size distribution was kept constant throughout the simulations. Fire regime parameters were set to change in 1830, 1890 and 1940 according to the different fire regime periods considered.

#### Simulations of timber harvest

Harvesting was performed using the Biomass Harvest extension in LANDIS-II (Gustafson et al. 2000). It is impossible to perform harvesting simulations in LANDIS-II based on dbh; rather, harvesting is performed based on biomass harvested over a specific area per timestep. As such, we calculated proxies to determine the percentage of cells affected for each species during selective cutting for square timber and diameter-limit cutting for sawn timber. We first calculated the historical volume harvested over an area equivalent to our study area for each species for a given time period. We converted these volumes to mass in tons using the green wood density of each species. Using accumulation curves, which plot cumulative biomass against the number of cells sampled, we determined the number of harvestable cells equivalent to the harvested biomass and the percentage of affected cells for each species. These were used to know the extent to which prescriptions in the Biomass Harvest extension had to be performed at each timestep. We identified four different harvesting periods: 1860–1890, 1890–1930, 1930–1990 and 1990–2000. These periods correspond to selective cutting, diameter-limit, clear-cut and modern harvesting eras, respectively. During the selective cutting era, harvesting was parameterized to be limited to white and red pine stands that were at least 200 years and 150 years old, respectively. Selective logging of white and red pines was performed at a rate of 18.7% and 0.124% of the landscape per timestep, respectively, during that era. During the diameter-limit era, two types of prescription mimicking diameter-limit harvest were performed. The first type targeted stands that included either white or red pines, with a minimum age of 70 years and 130 years, respectively. These prescriptions were performed on 20.29% and 12.63% of the landscape per timestep, respectively. The second type of diameter-limit harvest was performed in stands including balsam fir, yellow birch, white spruce, aspen and eastern hemlock, with minimum ages of 50, 50, 80 and 60 years, respectively, and was performed on 2.2% of the landscape per timestep. We also accounted for the fact that there was still selective logging of white and red pines during this era at reduced rates of 13.15% and 0.0004%, respectively. For the clear-cutting era (1930–1989), stands had to be at least 60 years old to be eligible for harvest and all cohorts were harvested when selected. According to historical rates, clear-cutting was performed on 22.2% of the territory at each timestep. For the 1990–2000 period, we applied partial-cutting at a 30% rate per timestep for which 25% of the biomass is removed for all species. For all prescriptions, the minimum time between harvest in a given stand was set to 40 years while patch size was restrained to one cell (6.25 ha).

In simulating historical timber harvesting, we did not include plantations, fertilization, thinning, or other forms of intensive forest management.

#### Other disturbances included in the simulations

We accounted for two additional natural disturbances, budworm outbreaks and catastrophic wind events. Both disturbances were included in all simulations as background factors. Spruce budworm (*Choristoneura fumiferana*), known to affect our study area (Bouchard et al. 2005, 2006a, 2006b) was simulated using the base biological disturbance agent (BDA) extension (Sturtevant et al. 2004). To reflect historical outbreak patterns, we set the interval between budworm outbreaks to 40 years. This corresponds to the mean interval observed over the last 450 years in the Bas-Saint-Laurent region of southeastern Quebec (Boulanger and Arseneault 2004), which includes areas with the same bioclimatic domain as in this study. For the windthrow disturbance, we used the Base Wind extension (Mladenoff and He 1999) to simulate wind events with a recurrence interval of 2500 years.

#### Modelling scenarios

**Fig. 2:**
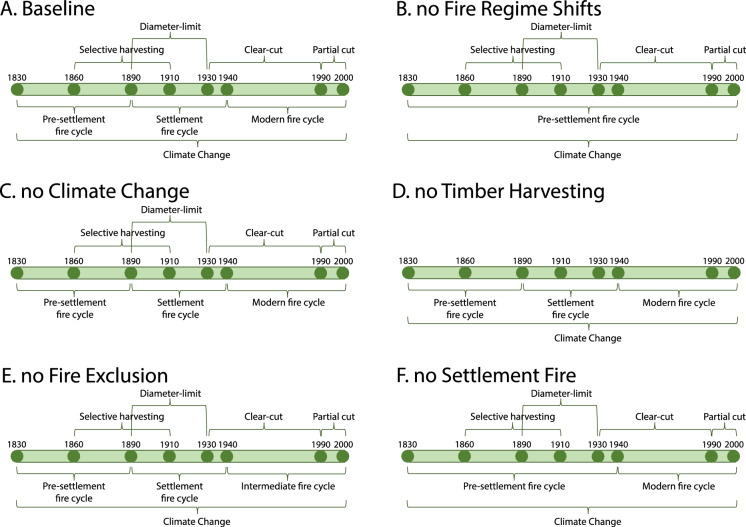
Timeline of all scenarios simulated: **A** Baseline scenario (BL) that included all disturbances that historically affected the landscape, **B** Succession without Fire Regime Shifts scenario (noFRS), **C** Succession without Climate Change scenario (noCC), **D** Succession without Timber Harvesting scenario (noTH), **E** Succession without Fire Exclusion scenario (noFE), **F** Succession without Settlement Fire scenario (noSF)

We modelled six scenarios, all spanning from 1830 to 2000 (Fig. 2) with five simulation replicates each. We ended the simulations in year 2000 to align with the 2001 Canadian National Forest Inventory (NFI), which provided the most comprehensive and relevant data for validating our retrospective model (see Analysis section below). We used a 10 year timestep. The baseline scenario included all historical disturbances and served as the reference scenario. To test H1, we isolated the individual effects of fire regime shifts, climate change, and timber harvesting on vegetation changes by creating three counterfactual scenarios, each excluding one of these disturbances. The scenario that excludes timber harvesting also addresses H2. To test H3, we included two additional counterfactual scenarios to isolate the effects of the fire regimes associated to fire exclusion and Euro-American settlement. The six scenarios were:

1. Baseline scenario (BL): this scenario included all disturbances that historically affected the landscape: fire regime shifts (pre-settlement, settlement, and modern), timber harvesting, climate change, background epidemics and catastrophic wind disturbances.
2. Succession without Fire Regime Shifts scenario (noFRS): this counterfactual scenario isolated the effect of shifting fire regimes by maintaining the pre-settlement fire regime throughout the entire simulation period.
3. Succession without Climate Change scenario (noCC): this counterfactual scenario isolated the effect of climate change by keeping climate conditions constant throughout the entire simulation.
4. Succession without Timber Harvesting scenario (noTH): this counterfactual scenario isolated the effect timber harvesting by excluding all logging activities during the entire simulation. Thus, we evaluated the historical evolution of timber harvesting practices as a single factor in our study.
5. Succession without Fire Exclusion scenario (noFE): This counterfactual scenario isolated the effect of the fire exclusion era by replacing the modern fire regime with slightly lower fire activity than the pre-settlement fire regime to account for wetter conditions in the most recent period.
6. Succession without Settlement Fire scenario (noSF): This counterfactual scenario isolated the effect of increased fire activity during to settlement activities by maintaining the pre-settlement fire regime during the period of colonization.

### Analysis

To validate our retrospective landscape model, we assessed whether the simulated forest vegetation for the year corresponding to 2000 AD in the BL approximated the modern forest vegetation. We constructed forest composition matrices based on the total aboveground biomass of each species within our landscape. The forest composition matrix for the LANDIS output was derived by averaging the five replicates of the BL. The matrix representing true modern conditions was derived from the Canadian National Forest Inventory (NFI) for year 2001. We employed the Bray–Curtis index to quantify the compositional differences between the simulated and the observed forest vegetation. We obtained the Beta diversity indices using the function *beta.pair.abund* from the R package *betapart* (Baselga et al. 2018)*.*

To assess the impact of the different disturbances on vegetation change, we performed a PERMANOVA (Permutational Multivariate Analysis of Variance) on the forest composition matrices based on the total aboveground biomass per species. The analysis compared the BL with each of the counterfactual scenarios. We used Bray–Curtis dissimilarities to calculate pairwise distances in the PERMANOVA between the BL and each counterfactual scenario for each time step of the simulation and reported the resulting R^2^ values. We performed these analyses using the *adonis* function of the *vegan* package in R (Oksanen et al. 2013).

## Results

### Validation of LANDIS-II model

The output of the BL for the year 2000 AD closely approximated contemporary vegetation conditions, as indicated by a turnover component of the Bray–Curtis dissimilarity of 0.32, a nestedness component of 0.10, and an overall Bray–Curtis dissimilarity of 0.41 (Table 3). The model overestimated aspen biomass while underestimating yellow birch.

**Table 3:** Abundance-based Bray–Curtis dissimilarity and its components for the comparison of the simulated landscape forest composition under the Baseline scenario for year 2000 and the observed forest composition from the national inventory for the year 2001

Metric	Value
Bray–Curtis Dissimilarity	0.41
Turnover	0.32
Nestedness	0.10

### Evolution of the landscape composition under the baseline scenario

**Fig. 3:**
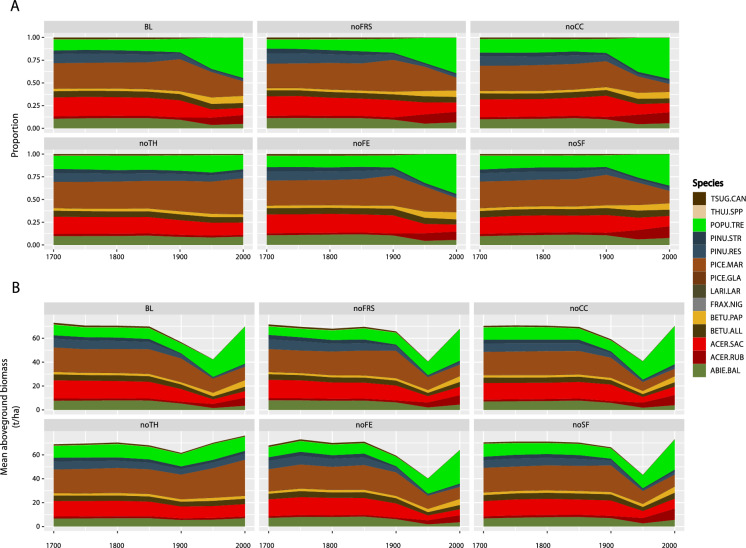
**A** Relative abundance of each species over time and **B** Cumulative biomass over time under the baseline scenario (BL) and each of the counterfactual scenarios: without fire regime shifts scenario (noFRS), without climate change scenario (noCC), without timber harvesting scenario (noTH), without fire exclusion scenario (noFE), without settlement fire scenario (noSF)

Total biomass under the BL varied throughout the simulated time period, mostly reflecting different harvesting eras. Total biomass remained relatively stable until 1890, after which it sharply dropped, reaching its lowest point around 1950, followed by a gradual recovery (Fig. 3). Species composition varied throughout the studied period. For instance, early successional, deciduous species such as aspen, red maple, and paper birch exhibited substantial increases in their abundance after 1900 that intensified around 1950. Specifically, aspen’s relative abundance increased from ~ 13 to 44%, and its cumulative biomass from 9 to 30 tons/ha (Fig. 3). Red maple’s relative abundance increased from ~ 3 to 13%, and its cumulative abundance from 2 to 8 tons/ha (Fig. 3). Paper birch’s relative abundance increased from ~ 3 to 8%, and its cumulative abundance from 3 to 5 tons/ha (Fig. 3).

Conversely, mid- and late successional species such as pines, spruce, balsam fir, and sugar maple experienced declines over time under the BL scenario. The pines’ relative abundance decreased from 13% in 1850 to 3% in 2000, with their cumulative abundance decreasing from 10 to 2 tons/ha (Fig. 3). Black spruce’s relative abundance decreased from 32% in 1850 to 18% by 2000, and its cumulative abundance decreased from 20 tons/ha in 1900 to 11 tons/ha by 2000 (Fig. 3). Balsam fir’s relative abundance decreased from 12.5% in 1850 to 4% by 2000, and its cumulative abundance decreased from 8 tons/ha in 1850 to 2 tons/ha in 1950, then recovering slightly to 4 tons/ha by 2000 (Fig. 3). Sugar maple’s relative abundance decreased from ~ 17% in 1850 to 8% by 2000, and its cumulative abundance decreased from 25 tons/ha in 1850 to around 5 tons/ha by 2000 (Fig. 3).

### Relative impacts of timber harvesting on forest composition

**Fig. 4:**
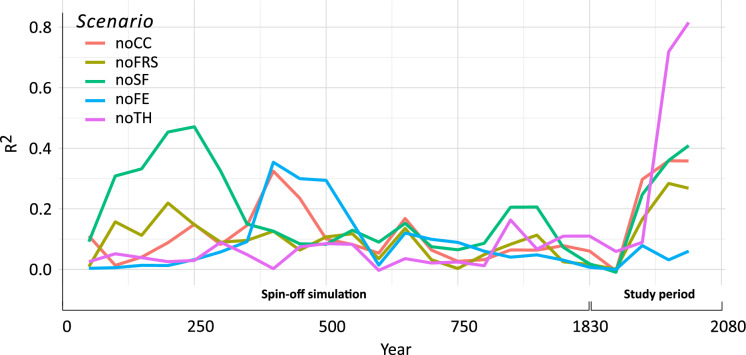
PERMANOVA of the forest composition matrices between the historical baseline scenario (BL) and each of the counterfactual scenarios: without fire regime shifts scenario (noFRS), without climate change scenario (noCC), without timber harvesting scenario (noTH), without fire exclusion scenario (noFE), without settlement fire scenario (noSF). Shown is the spin-off simulation of 1000 years followed by our study period (1830–2000)

The noTH, controlling for timber harvesting, showed the greatest deviation from the BL in vegetation composition among all the counterfactual scenarios. It had the highest R^2^ values in the PERMANOVA analysis, reaching 0.70 around 1950 and 0.80 by 2000 (Fig. 4). Unlike the BL and the other counterfactual scenarios, the noTH showed relatively stable species abundance over time (Fig. 3). However, the noTH showed an increase in black spruce and a decrease in red maple. Specifically, the relative abundance of black spruce increased from 26% in 1900 to 38% by 2000, and its cumulative abundance increased from 20 to 30 tons/ha (Fig. 3). In contrast, red maple’s relative abundance decreased from 19% in 1850 to 13% in 2000, and its cumulative abundance decreased from 12 to 10 tons/ha (Fig. 3). Aspen exhibited a temporary increase from 16% in 1900 to 18% in 1950, but declined to 16% by 2000, resulting in no net change by the end of the simulation (Fig. 3A). Similarly, aspen’s cumulative abundance increased from 10 tons/ha in 1900 to 14 tons/ha in 1950 but decreased to 11 tons/ha by 2000 (Fig. 3B).

### Relative impact of fire regimes on forest composition

The noFE, controlling for fire exclusion, showed minimal divergence in vegetation composition from the BL, with R^2^ values from the PERMANOVA consistently remaining below 0.10 throughout the study period (Fig. 4). In contrast, the noSF, controlling for settlement increased fire activity, exhibited the highest divergence after the noTH, with R^2^ values increasing from 0.25 in 1930 to 0.40 by 2000 (Fig. 4). Similarly, the noFRS, controlling for fire regime shifts, showed an increase in R^2^ beginning in 1900 reaching 0.30 by 2000 (Fig. 4). The changes in species biomass under the noFRS, noFS, and noFE closely resembled those observed under the BL. Under the noSF, the relative and cumulative abundances of aspen increased after the 1900s similarly to what was simulated under the BL; however, the increase was smaller than under the BL yet greater than under the noTH (Fig. 3).

### Relative impact of climate change on forest composition

The noCC, controlling for climate change, exhibited modest divergence from the BL in forest composition, as indicated by R^2^ values in the PERMANOVA analysis that increased from 0.20 in 1860 to 0.30 by 1930 and reached 0.35 by 2000 (Fig. 4). The overall evolution of species composition under the noCC closely resembled that of the BL. However, the decline in black spruce observed under the BL was slightly more pronounced under the noCC.

## Discussion

This study represents the first retrospective modelling of forest dynamics and disturbances in the mixedwood temperate forests of southwestern Quebec. It demonstrated the enduring influence of past forest use, as these legacies have shaped distinct successional trajectories and still influence the regional forest composition. Although previous studies based on historical land survey records have reported the transformation in the forest composition from pre-settlement to settlement times in the region (Jackson et al. 2000b; Danneyrolles et al. 2016a), they have not examined the individual effects of various disturbances on driving these shifts.

Our results suggested that timber harvesting had the greatest impact on dynamics of vegetation composition, supporting H1. This is consistent with the extensive and intense historical timber harvesting in the region. In the absence of timber harvesting (noTH), biomass abundance for most species remained relatively stable throughout the simulation, indicating ecosystem resilience and supporting H2. The settlement-related increased fire activity had a modest effect on the vegetation composition, while fire exclusion had a minimal effect, rejecting H3 and indicating that the effects of fire exclusion on mesophication is a slow, gradual process that may take longer time to observe. While the direct effects of climate change were not as pronounced as the impacts of timber harvesting, due to the modest climate change observed during the study period, it still contributed to the dynamics observed in black spruce.

Our conclusions relied on a well-verified baseline scenario (BL) that aligned closely with observed data from the national inventory for the present day. The evolution of aboveground biomass simulated by the BL was consistent with the findings from previous studies in the region (Jackson et al. 2000a; Friedman and Reich 2005; Schulte et al. 2007; Dupuis et al. 2011a; Danneyrolles et al. 2016a; Terrail et al. 2019). The successful performance of the BL scenario underscored the reliability of pre-settlement vegetation and historical fire regime reconstructions in the area.

### Evolution of vegetation composition in the baseline scenario

The BL scenario revealed a significant increase in fast-growing broadleaf species, including aspen, paper birch, and red maple, alongside a decline in late-successional, long-lived conifers such as pines, spruce, and balsam fir, but also a decrease in sugar maple, a broadleaf, late-successional species over the last 170 years (Fig. 3, Table 4). In general, these results were consistent with previous studies of forest dynamics from pre-settlement to modern times (Jackson et al. 2000a; Friedman and Reich 2005; Schulte et al. 2007; Dupuis et al. 2011a; Danneyrolles et al. 2016a; Terrail et al. 2019), validating our simulation and supporting the evaluation of the counterfactual scenarios.

**Table 4:** Comparison of composition changes per species in the baseline scenario of this study and those reported by Danneyrolles et al.’s (2016a)

Species	This study’s baseline scenario	Danneyrolles et al. (2016a)	
	Relative abundance % change (%)	Dominance % change (%)	Prevalence % change (%)
Spruces (*Picea*)	− 43.75	− 59.35	− 8.19
Basam fir (*Abies balsamea*)	− 68.00	− 37.50	+ 0.43
Pines (*Pinus*)	− 76.92	+ 53.85	− 23.17
Poplars (*Populus*)	+ 238.46	+ 228.74	+ 155.65
Paper birch (*Betula papyfera*)	+ 166.67	+ 110.96	+ 19.93
Red maple (*Acer rubrum*)	+ 333.33	+ 3000	+ 4272.73
Sugar maple (*Acer saccharum*)	− 52.94		

Dominance % is the percentage of each taxon occurring in the first rank of enumeration in the taxon lists. Prevalence % is the percentage occurrence of each taxon in all taxon lists, regardless of its rank in those lists (See Danneyrolles et al. 2016a for further details on these values)

The increase in the broadleaved species was largely due to their ability to thrive in disturbance-prone environments. Aspen’s relative abundance tripled during the twentieth century, rising from 13% in 1900 to around 44% in 2000 (Fig. 3A). Aspen is able to regenerate vegetatively through root suckering when the aboveground portion of the tree is removed or damaged (Frey et al. 2003). Normally, aspen roots do not develop suckers when intact aboveground parts are present, as auxins hormones that inhibit sucker initiation are transported to the root system (Wan et al. 2006). When the aboveground portion of the tree is removed, the lack of auxins allows cytokines, signaling proteins produced in the roots, to promote the development of stem buds and shoots (Wan et al. 2006). This vegetative strategy enables aspen to quickly colonize open spaces following disturbances, particularly when a large portion of the basal area is removed (Prévost and Pothier 2003). Furthermore, increased soil temperatures on disturbed sites have been shown to facilitate re-sprouting (Frey et al. 2003).Paper birch more than doubled in relative abundance, increasing from 3% in 1900 to 8% by 2000 (Fig. 3A). Paper birch, like aspen, thrives under disturbances due to its ability to reproduce vegetatively, resprouting from the base or roots when cut or damaged. Both sprouting or seeding of paper birch are abundant, enabling it to dominate in the absence of aspen (Bergeron 2000). Red maple, while not strictly a shade-intolerant pioneer or fire-adapted species (Nowacki and Abrams 2008) like aspen and paper birch, showed a remarkable fourfold increase in relative abundance, from 3 to 13% (Fig. 3A). This increase is due to its status as a “super generalist” favored by disturbances and having shade tolerance (Abrams 1998). Red maple is one of the fastest growing trees in the region (Zhang et al. 2015), growing rapidly after germination, maturating early and producing abundant seeds (Abrams 1998). Red maple seedlings can persist under shaded conditions, but quickly increase growth in response to gap openings (Abrams 1998). This adaptability allows red maple to thrive both in understory and open environments (Archambault et al. 2009).

The decline in conifers is explained by early timber harvesting specifically targeting them. The relative abundance of pines had a fourfold decrease, going from 13% in 1850 to 3% by 2000 (Fig. 3A). This decline started earlier than the decrease of the other conifers, 1850 compared to 1900. The earlier decrease coincides with the square timber harvesting period (1860–1910), during which the largest pines were targeted, resulting in the extraction of a total of *ca* 138 kilotons of white and red pine combined across our landscape (Table 1).

The decline in conifers can also be attributed to competition with the fast-growing broadleaved species that expanded following intensive cutting. As slow growers, conifers take longer to reach sexual maturity, making species like black spruce particularly vulnerable when disturbance occur at short intervals (Brown and Johnstone 2012). This vulnerability likely contributed to a decrease in black spruce’s relative abundance from 32 to 18% (Fig. 3A). As a fire-adapted species, black spruce relies on its aerial seed bank, storing its seeds in semi-serotinous cones. These cones typically remains closed until a fire melts the sealing resin, triggering seed release and enabling a regeneration pulse (Brown and Johnstone 2012; Viglas et al. 2013). Black spruce trees may produce cones by age 30 but are more likely to do so by age 100 (Viglas et al. 2013). If fires or other disturbances occur too frequently before the trees have matured and produced sufficient cones, it can severely compromise recruitment.

The decline in sugar maple, with a relative abundance of 17% in 1850 that dropped to 8% by 2000, can be attributed to its status as a late-successional species (Barrette et al. 2024). However, this decrease does not agree with previous research that have reported an increase in both red maple and sugar maple from pre-settlement to modern times in the region (Dupuis et al. 2011a; Danneyrolles et al. 2016a). Notably, Danneyrolles et al. (2016a) conducted their study in Temiscamingue and used the same dataset as in this study. The discrepancy might be due to model idiosyncrasies, particularly the life trait parameters we used in our simulations, which more closely follow sugar maple’s late-successional status in this region. While the increase in sugar maple has been explained by its high phenotypic plasticity and sprouting ability after disturbances (Nolet et al. 2008), these traits are known to vary with latitude (Nolet et al. 2008).

### Controlling for timber harvesting

Timber harvesting emerged as the primary driver of the vegetation shifts observed, supporting H1. The counterfactual scenario that excluded timber harvesting (noTH) deviated the most from the BL, with an R^2^ of 0.85 by 2000 (Fig. 4). Under the noTH, the aboveground biomass of most species remained relatively stable in contrast to the more pronounced shifts observed under the BL and other counterfactual scenarios (Fig. 3). However, there were some exceptions. For instance, black spruce increased, while sugar maple decreased. Aspen exhibited a temporary increase but later declined, resulting in no net change by the end of the simulation.

Timber harvesting focusing on a particular set of canopy dominants likely overrode effects of other factors in shaping vegetation of the studied landscape. A similar pattern was reported in another study in the forests of central Ontario (Jackson et al. 2000b). The decrease of spruce and pine in eastern Quebec (Dupuis et al. 2020), and the widespread increase in red maple in the region (Fei and Steiner 2009) have been largely attributed to logging. Projections of forest dynamics under future climate change have equally identified timber harvesting as an important driver of changes in mixedwood boreal forests of eastern Canada (Molina et al. 2021). Our findings corroborate these conclusions. However, changes in the abundance of certain species have also been attributed to other factors. For example, the increase in aspen in eastern Quebec has been attributed to increased fire activity related to settlement activities (Dupuis et al. 2020).

By removing timber harvesting from our simulations (noTH), we allowed the forests to experience only natural disturbances particularly during the last 60 years of the simulation (1940–2000), when settlement-related fire activity ended. Under the noTH scenario, after 1940, disturbances were limited to budworm outbreaks and windthrow. These partial disturbances, along with small gap dynamics, could have promoted both constant and pulse recruitment (Després et al. 2014). Intermediate disturbances may have facilitated the persistence of early-successional species, like aspen and paper birch (Kneeshaw and Bergeron 1998), a pattern observed in old temperate hardwood forests under natural disturbance regimes in the region (Frelich and Reich 1995). The modest increase in the relative biomass of the shade-tolerant black spruce (from 26% in 1900 to 38% by 2000), could be attributed to black spruce’s lower susceptibility to budworm outbreaks, allowing it to expand following the decline of balsam fir.

The relatively stable biomass of all species throughout the simulation suggests that ecosystem resilience was maintained, supporting H2. In the absence of timber harvesting, functional traits present during the pre-settlement period such as fire tolerance and shade tolerance were preserved. The removal of species with key functional traits, such as fire tolerance, reduces the system’s ability to recover and increases its vulnerability to novel disturbances (Johnstone et al. 2016; Seidl et al. 2016).

### Controlling for fire regimes

The effect of the settlement-related increased fire activity on the forest composition, although modest, was greater than that of fire exclusion. Controlling for settlement fires (noSF), resulted in the second highest values of dissimilarity from the BL, with an R^2^ that reached 0.40 by 2000 (Fig. 4). Although the biomass evolution under the noSF was not markedly different from the BL, the increase in aspen was less pronounced. This finding aligns with the increase in aspen being driven by higher fire activity during the settlement period (Dupuis et al. 2020). However, our study suggests that settlement-related increased fire activity was only part of the story, with logging playing the dominant role.

In contrast, modern fire exclusion (noFE) had the lowest impact on vegetation composition, with R^2^ values consistently below 0.10 throughout the simulation (Fig. 4), and biomass changes closely mirroring those in the BL. This contradicts H3 and previous research that identified fire exclusion as the main driver of forest mesophication, where fire-sensitive mesophytic species like maple and birch increase while fire-dependent xerophytic species like pine decline (Nowacki and Abrams 2008; Frelich et al. 2021). This discrepancy may be due to the mesophication process likely unfolding over longer timescales than those captured by this study. Unpublished data provided by Stathopoulos (Theodore Stathopoulos, pers. comm.) indicate that the present recruitment of red pine, a fire-adapted species, in our study area is poor to non-existent. Red pine’s regeneration in these forests was largely confined to periods before fire exclusion, with most tree ring piths dating to the late 1800s and early 1900s. The more pronounced effect of settlement-related fires (noSF) compared to fire exclusion (noFE) can be attributed to the immediate impacts of increased fire activity, which promotes the rapid establishment of early-successional species. In contrast, the effects of fire exclusion are more gradual, with a slow replacement of fire-adapted species by fire-sensitive ones over time.

### Controlling for climate change

Direct effects of climate change had a modest impact on the forest composition. When climate change was controlled for (noCC), the R^2^ value reached 0.35 by the end of the simulation in 2000. The biomass evolution largely mirrored that of the BL. However, the decline in black spruce was slightly more pronounced under the noCC compared to the BL and the other counterfactual scenarios, suggesting that climate change in our study area favored the growth of black spruce. A positive growth response for black spruce has been particularly observed in the eastern, wetter regions of North America, including our study area (Wang et al. 2023). The parameters used to model black spruce growth in our simulations were informed by Wang et al.’s (2023). In contrast, Girardin et al. (2016) projected that black spruce at the southern edge of its distribution, where temperatures are higher, experiences reduced growth due to increased respiratory demand. Nevertheless, the concurrent increase in mean annual precipitation of 1.66% in our study area could mitigate evapotranspiration and positively influence black spruce growth. In forests located south of 49°N latitude, higher solar radiation leads to increased evapotranspiration, resulting in a more moisture-limited environment compared to northern forests (Lesven et al. 2024).

The scenarios controlling for fire exclusion (noFE) and for the increased fire activity during the settlement period (noSF) partly reflected the indirect effects of climate change. Fire exclusion was influenced by increased moist conditions in the area (Girardin et al. 2006; Drobyshev et al. 2017; Chavardès et al. 2022). However, the noFE scenario had the lowest impact on the vegetation composition among all the counterfactual scenarios (Fig. 4), suggesting that the indirect effects of climate change through fire exclusion during our study period were minimal. Similarly, increased fire activity during the settlement period was affected by climate, as drought conditions during 1910–1920 contributed to higher fire hazard (Girardin et al. 2009). The noSF had the second strongest effect on the vegetation composition (Fig. 4) and contributed to some extent to the increase of aspen (see Controlling for fire regimes section above). However, the extent to which these effects were driven by indirect climate influences remains uncertain.

The relatively modest impact of climate change on vegetation compositions changes from the pre-settlement to the present aligns with research showing that increased precipitation in this area over the past century has offset rising temperatures (Girardin et al. 2009, 2013). This has helped prevent the modern record drought-induced declines in forest productivity and diebacks seen in boreal forests in western Canada and Alaska (Hogg and Bernier 2005; Soja et al. 2007), and the increased fire activity in northwestern North America (Hanes et al. 2018; Chavardès et al. 2022). However, the future effects of climate change on the forest composition, whether directly or through climate-sensitive disturbances, in the area remain uncertain. Projections under the AR4 A2 scenario suggest that eastern North America will continue receiving enough precipitation to offset increased evapotranspiration in 2090 (Gauthier et al. 2015). Meanwhile, under the RCP 8.5 scenario, climate change effects on tree growth, competition, and area burned are projected to cause drastic shifts in forest composition by 2100 (Boulanger et al. 2018).

### Study limitations

We acknowledged several limitations of this research. The primary limitation is related to the challenges of precisely recreating the pre-settlement vegetation conditions and accurately replicating the timing of the disturbances. Even though our retrospective simulations showed overall meaningful trends, our inability to perfectly reconstruct historical conditions that led us to employ several simplifying assumptions in the simulations introduced some uncertainty that affects the interpretation of our findings.

We did not test interactions between disturbances. Our experimental design focused on isolating the effects of single disturbance types. However, disturbances can have interactive effects when occurring successively in a stand (Kulakowski et al. 2003; Buma 2015).

### Implications for southwestern Quebec’s temperate hardwood forests

Our study shows that legacies from past forest use, primarily timber harvesting but also settlement-related increased fire activity, have altered the vegetation composition of southwestern Quebec’s forests, leading to a decline in long-lived fire-adapted conifers, like red pine, and the rise of early successional broadleaf trees, like aspen. With climate change projected to further alter forest composition, current management should prioritize restoring forests with a diversity of functional traits to enhance their resilience to future disturbances (Boulanger et al. 2018; Lucash et al. 2018). Increasing species and functional diversity can enhance ecosystems’ response flexibility to changing conditions, helping maintain ecosystem functioning and services under changing environmental conditions (Mori et al. 2013; Seidl et al. 2016). Disentangling the effects of different global change-related drivers of forest ecosystem dynamics will remain a key research topic to ensure that forests can provide their essential ecosystem functions and services, including biodiversity, carbon storage, and wood production.

## Supplementary Information

Below is the link to the electronic supplementary material.

Supplementary file1 (DOCX 38 KB)

## Data Availability

Data are available upon reasonable request.

## Abbreviations

LIA: Little ice age
MWP: Medieval warm period
AGB: Aboveground biomass
BL: Baseline scenario
noFRS: Without fire regime shifts scenario
noCC: Without climate change scenario
noTH: Without timber harvesting scenario
noFE: Without fire exclusion fire regime scenario
noSF: Without settlement fire scenario
