# Supplementary material for: Timber harvesting was the most important factor driving changes in vegetation composition, as compared to climate and fire regime shifts, in the mixedwood temperate forests of Temiscamingue since AD 1830
Source: Landsc Ecol. 2025 Jan 20;40(2):26. doi: 10.1007/s10980-025-02043-x (PMC11753300; doi:10.1007/s10980-025-02043-x)
Supplement: Supplementary file 1 — Supplementary file1 (DOCX 38 KB) [file 10980_2025_2043_MOESM1_ESM.docx]

Supplementary Information for:

Timber harvesting was the most important factor driving changes in vegetation composition, as compared to climate and fire regime shifts, in northeastern North American temperate forests since AD 1830

*Landscape Ecology*

Daniela Robles, Yan Boulanger, Jesus Pascual, Victor Danneyrolles, Yves Bergeron, Igor Drobyshev

Igor Drobyshev is the corresponding author, [igor.drobyshev@slu.se](mailto:igor.drobyshev@slu.se)

Supplementary Information 1.

Timber Harvest Data Processing

We obtained the harvest data from the annual reports of the commissioners of the crown lands for Quebec, which later became the Ministry of Lands and Forests of Quebec, retrieved from the Library of the National Assembly of Quebec (<https://www.bibliotheque.assnat.qc.ca//fr/>). These reports included the surface area under license for the timber industry and the annual extracted volume for each species across different regions. We used data for the Upper Ottawa Valley region because Temiscamingue is located within the broader Upper Ottawa region, and data specifically for Temiscamingue were unavailable in these reports. We assumed that the extracted volume per surface area unit for each species in the Upper Ottawa valley was uniform across the region.

We distinguished four distinct timber harvesting phases: (a) selective cutting of large white and red pines for square timber during 1860-1910; (b) diameter-limit cutting of white and red pines, and cuttings of at least three species among white spruce, balsam fir, eastern hemlock, yellow birch, and aspen for sawn timber during 1890-1930; (c) clear-cutting of all species during 1930-1990 and (d) partial-cutting of all species during 1990-2000.

For (a), the extraction focused on the largest pines with a diameter at breast height (dbh) above 50.8 cm, corresponding to 200+ year old white pines (Pinchot and Graves 1896) and 150+ year old red pines (Kipfmueller et al. 2021). For (b), we determined the minimum harvesting diameter for each species based on government regulations established in 1909. For the trees to be cut, these regulations recommended the minimum diameter at the stump (dsh), which we converted to dbh using the conversion table from the Direction of forestry inventories’ report (Direction des inventaires forestiers 2005). The resulting minimum dbh values were: 23.88 cm for white pine, 24.45 cm for red pine, 20.92 cm for white spruce, 17.56 cm for balsam fir, 17.83 cm for hemlock, 16.94 cm yellow birch, and 18.36 cm for aspen. These diameter limits roughly corresponded to the following age classes : ≥ 90 years for white pine (Pinchot and Graves 1896), ≥ 130 years for red pine (Kipfmueller et al. 2021), ≥ 45 years for white spruce (Burgar 1961), ≥ 45 years for balsam fir (Burgar 1961), ≥ 60 years for hemlock (Lorimer 1980), ≥ 50 years for yellow birch (Lorimer 1980), and ≥ 80 for aspen (Nunifu 2009). However, site conditions cause variation in dbh among trees of the same age (Chen et al. 2020), and these estimates are rough approximations.

For years where the commissioners’ reports were missing, we interpolated extracted volumes by averaging the annual extracted biomass of previous and subsequent years. The commissioners’ reports combined the harvested volume of red and white pine for square timber from 1900-1910. For this period, we assumed that the percentages of white and red pine volume were the average percentages for the 1860-1899 period, 91.23% and 8.77%, respectively.

The harvested volume for sawn timber in the commissioners’ annual reports presented some inconsistencies. The unit of measurement for sawn timber varied over time, but we converted them to cubic feet using conversion tables in Gaudreau’s (1986) and Ortuno et al.’s (2010). The reported volumes of white pine, red pine, and white spruce for sawn timber included combinations with other species. Considering that white pine, red pine, and spruce were the primary species of interest for sawn timber, it is reasonable to assume that a significant portion of the reported combined volumes consisted primarily of these three species. We used the volumes given by the reports for white and red pine, acknowledging the wasteful logging practices of the time that resulted in a significant volume being left behind in logged areas (Doyle 1952). However, for white spruce, the pixels harvested had to include a combination of ≥ 3 species of white spruce, balsam fir, eastern hemlock, yellow birch, or aspen because the available biomass of white spruce alone in our landscape was insufficient to account for the extracted biomass reported. Despite some inconsistencies and necessary assumptions, these reports present the most comprehensive and official source of information available for historical logging.

References

Burgar RJ. 1961. The relative growth rates of white spruce and balsam fir trees in the port arthur district of ontario. For Chron. 37(3):217–223. doi:10.5558/tfc37217-3.

Chen J, Yang H, Man R, Wang W, Sharma M, Peng C, Parton J, Zhu H, Deng Z. 2020. Using machine learning to synthesize spatiotemporal data for modelling DBH-height and DBH-height-age relationships in boreal forests. For Ecol Manag. 466:118104. doi:10.1016/j.foreco.2020.118104.

Direction des inventaires forestiers. 2005. Relations entre le diamètre à hauteur de poitrine (DHP) et le diamètre à hauteur de souche (DHS) pour les principales essences commerciales du Québec. Ministère des Ressources naturelles et de la Faune. [accessed 2023 Jun 20]. https://mffp.gouv.qc.ca/nos-publications/relations-dhp-dhs-essences-commerciales/.

Doyle J. 1952. Good Logging Practices Increase Forest Yields. For Chron. 28(4):36–41.

Gaudreau G. 1986. L’exploitation des forêts publiques au Québec, 1842-1905. Presses Université Laval.

Kipfmueller KF, Larson ER, Johnson LB, Schneider EA. 2021. Human augmentation of historical red pine fire regimes in the Boundary Waters Canoe Area Wilderness. Ecosphere. 12(7):e03673. doi:10.1002/ecs2.3673.

Lorimer CG. 1980. Age structure and disturbance history of a southern Appalachian virgin forest. Ecology. 61(5):1169–1184.

Nunifu TK. 2009. Compatible diameter and height increment models for lodgepole pine, trembling aspen, and white spruce. Can J For Res. 39(1):180–192.

Ortuno M, Doyon E, Jean F. 2010. Distribution historique du pin blanc et rouge en Outaouais - Phase 2 - Évaluation de la quantité exploitée dans les forêts publiques au 19e siècle. Ripon, Québec: Institut québécois d’Aménagement de la Forêt feuillue.

Pinchot G, Graves HS. 1896. The White Pine: A Study, with Tables of Volume and Yield. Century Company.
